# Genetic Variations of Tumor Necrosis Factor –α-308 and Lymphtoxin-α+252 in Non-Hodgkin Lymphoma and Acute Lymphoblastic Leukemia Patients

**Published:** 2013-09

**Authors:** Hajar Nasiri, Safar Farajnia, Azim Rezamand, Ali Akbar MovassaghPour, Heydar Ali Esmaeili, Amir Monfaredan, Naser Mobarra, Nasser Rahimifar, Leyla Sahebi, Majid Farshdousti Hagh

**Affiliations:** 1 School of Medicine, Tabriz University of Medical Sciences, Tabriz, Iran; 2 Biotechnology Research Center, Tabriz University of Medical Sciences, Tabriz, Iran; 3 Childeren Hospital, Tabriz University of Medical Sciences, Tabriz, Iran; 4 Tabriz branch, Islamic Azad University of Medical Science, Tabriz, Iran; 5 Clinical Biochemistry Department, School of Medicine, Tehran University of Medical Sciences, Tehran, Iran; 6 Tuberclosis and Lung Disease Research Center, Tabriz University of Medical Sciences, Tabriz, Iran; 7 Hematology & Oncology Research Center, Tabriz University of Medical Sciences, Tabriz, Iran

**Keywords:** Acute lymphocytic leuke- mia, LT-α, Non-Hodgkin lymphoma, Polymorphism, TNF-α

## Abstract

***Objective(s): ***Non- Hodgkin lymphoma (NHL) and acute lymphoblastic leukemia (ALL) are two main hematological malignances which have been driven from lymphoid tissue. Genetic polymorphisms in tumor necrosis factor-α (TNF-α) -308 and lymphotoxin-α (LT-α) +252 may affect their transcription and expression which leads to their high plasma level. The frequency of the TNF-α (-308) and LT-α (+ 252) polymorphisms are different for NHL and ALL cases in various populations with different ethnicity. This research is designed to investigate the prevalence and association of TNF-α (-308) and LT-α (+ 252) polymorphisms from NHL and ALL in Azarian patients and healthy individuals from Northwestern part of Iran.

***Materials and Methods:*** Seventy subjects with ALL and 68 NHL, along with another 130 healthy subjects as control group took part in this study. Genomic DNA was extracted, then genetic polymorphisms in TNF-α and LT-α genes were analyzed with the PCR-RFLP and NCOI as restriction enzyme. A statistical analysis was performed by chi-square test using SPSS software. A *P*-value of <0.05 was considered statistically significant.

***Results:*** A statistically significant difference of LT-α polymorphism was in NHL patients and control (*P-*value= 0.008) but there was not any association of TNF-α polymorphism between NHL patients and control group. A significant association for TNF-a variant was in ALL and control (*P-*value =0.005), however, there was no relationship about LT variant between ALL and control.

***Conclusion:*** The results show that there are significant differences between TNF-α (-308) and LT-α (+252) genetic polymorphisms respectively in ALL and NHL patients with control group from Northwestern part of Iran.

## Introduction

Non-Hodgkin lymphomas (NHLs) are a large heterogeneous group of B- and T-cells lymphomas with uncontrolled malignant clonal expansion (1). They include a variety of clinicopathologic subtypes; each subtype has distinct epidemiology, immunophenotype, prognosis and, above all, response to therapy. About 90% of all NHLs cases are made of B-cell. Diffuse large B-cell lymphoma (DLBCL) and follicular lymphoma (FL) is two major of NHL subtypes (2-4). The etiologies of the most lymphomas remain unknown but some specific translocations and genetic variations are associated with cause of some NHL subtypes e.g. t(8:14) in Burkitt lymphoma (5). 

Acute lymphocytic leukemia (ALL) is the main type of leukemia in children. It is the most common childhood cancer all over the world (6), however, it affects adults too (7). ALL is a biologically and clinically heterogeneous disease and peak incidence of the disease is between two to five years old (6); but infantile lymphoblastic leukemia, a highly malignant biological subtype is common in one year old children and it has a poor prognosis (6). The majority of ALL cases are derived from B-cell progenitors, although, T-ALL is made of 10% all of cases. It is more severe than B-ALL and more common in adolescences (8). To date, the prevention and control of some diseases become more possible by the use of genetic factors as proper diagnostic and prognostic tests because they are always reliable and unchangeable. A group of genes that alter B cell survival and growth include regulatory and pro inflammatory cytokine genes    (1, 2). Tumor necrosis factor-α (TNF-α) and lymphtoxin-α (LT-α) are appropriate candidate genes for study of lymphoma and leukemogeneis because they code important immunoregulatory cytokines which are critical mediators of inflammation and apoptosis. They can act as autocrine growth factors in lymphoid tumors (9, 10). 

**Table1:** Demographic information of acute lymphoblastic leukemia patients

	Gender		Ethnic		Age	
	Male	Female	Azari	Others	Years (%)	
ALL patients	43	27	69	1	≤1	2.9
					2-5	38.6
					6≤	58.6
Diagnosis lineage	Pre B-cell		Pro B-cell		B-cell	T-cell
	45	9	1	14	1	
	64.3	12.9	1.4	20	1.4	

TNF-α and LT-α are pleiotropic cytokines of tumor necrosis factor family; both cytokines have similar biological activities (9). The genes coding for TNF-α and LT-α are located on chromosomal region 6p21.3-21.1. They are closely linked to HLA-B locus of major histocompatibility complex (9, 11). Genetic polymorphisms in TNF-α and LT-α locus affect expression level of their genes    (2). Exchanging guanine by adenine at position −308 in promoter region of TNF-α results in two allelic forms. The common type is TNF-G (TNF1) allele and another one is TNF-A (TNF2) allele (12, 13). A polymorphism in first intron of LT- α gene at position +252 (A→G) leads to two different alleles; the common allele is LT-A (10.5 kb) and LT-G (5.5 kb) is variant allele (12, 14). Variant alleles of TNF2 and LT-G (5.5 kb) have a strange transcriptional activation, it leads to their higher serum levels (14). NHLs and ALL patients with these polymorphic alleles have high plasma levels of TNF and LT. They have been associated with a poor prognosis, higher rate of relapse and shorter survival especially in DLBCL subtype of NHL. TNF- α and LT-α cytokine polymorphisms may affect autoimmune diseases such as rheumatoid arthritis, leukemia and lymphoma genesis by hinder DNA repair mechanisms or up-regulation of pro inflammatory and anti-apoptotic signals via nuclear transcription factor kappa B (NF-kB) pathway    (2). Over production of TNF-α and LT-α induce NF-kB pathway more than normal pattern. NF-kB performs two roles: First, it has anti-apoptotic properties, so it prevents cell death among cells with malignant potential. Second, it stimulates immune response, specifically production pro inflammatory cytokines which permit survival and proliferation of these cells (10, 15). 

This research was carried out to determine the prevalence of TNF-α (-308) and LT-α(+252) polymorphisms in NHL and ALL patients from Tabriz Children Hospital in Northwestern part of Iran.

## Materials and Methods

***Patients and healthy subjects***

This descriptive study was done on two patients groups: Seventy ALL and 68 NHLs patients from Tabriz Children Hospital and 130 healthy individuals as control group without any history of malignancy. Both groups prepared signed written consents for taking part in current study in conformity with Ethics Committee of Hematology and Oncology Research Center; Tabriz University of Medical Sciences. Demographic characteristic of ALL and NHL patients are summarized in Table 1 and Table 2.

Diagnoses of NHLs were based on lymph node biopsy excision from affected lymph nodes and conventional histopathological examinations. Immunohistochemical (IHC) studies (for some subjected patients) were done to confirm corrected diagnosis NHL subtypes. Samples related to Hodgkin lymphoma patients are omitted from the study. The staging of NHL was categorized according to Ann Arbor classification. Diagnosis of ALLs (B and T-All) was based on clinical valuation, complete blood count and bone marrow evaluation and confirmed by flowcytometry and cytogenetic analysis on random samples.

***Determination of polymorphisms with RLFP-PCR***

Five milliliter peripheral blood was taken in aseptic conditions from ALL patients and control group and DNA was extracted by standard conventional salting out- chloroform method.

**Table 2:** Demographic information of NHL patients

	Gender		Ethnic		Age	
	Male	Female	Azari	Others	Years (%)	
NHL patients	48	20	64	4	≤1	(32.7)
					11-50	(38.8)
					51≤	(28.6)
Subtypes	DLBCL	BL	FL	ALCL	N-C	
Frequency	35	6		7	10	
Percent	51.5	8.8	14.7	10.3	14.7	

NHL: Non hodgkin lymphoma. BL: Burkitt lymphoma. FL: Follicular lymphoma. ALCL: Anaplastic large cell lymphoma. N-C: Non categorized in special NHL subtype

DNA extraction from paraffin blocks of NHL patients were performed using DNA extraction kit (QIAGEN, QIAamp DNA FFPE Tissue Cat. No 56404) according to the manufacture, s instructions.

All PCR reactions were performed in a total volume of 20 µl containing 10 µl Master mix (Red Ampliqon, Cat No.190301), 0.5 µl forward primer (5 pmol), 0.5 µl reverse primer (5 pmol), 8 µl nuclease-free water and 1 µl Genomic extracted DNA. Genotyping for TNF-α(-308) (G→A) and LT-α(+252) (A→G) polymorphisms was performed by conventional polymerase chain reaction (Corbet CG1-96 serial: C070711) followed by restriction fragment length polymorphism (RFLP) analysis with NcoI Restriction enzyme and 1x Thermo scientific Tango Buffer (Fermentas, Cat. No. #ER0571). Primers used for amplification of a TNF-α promoter region are shown in Table 3. The applied TNF PCR program was at 95°C for 5 min, followed by 35 cycles of denaturation at 95°C for 20 sec, annealing at 58◦C for 20 sec, and extension at 72°C for 40 sec. A final extension step was carried out at 72°C for 7 min. The 107-bp fragment of TNF promoter region which included normal TNF-α allele (GG allele) at the nucleotide position -308 was digested by NcoI to 20 and 87 bp fragments whereas the TNF-α allele (AA allele) remained undigested (13).

The LT-α(+252) (A→G) polymorphism was analyzed by PCR amplification of a 371 bp fragment using primers that were shown in Table 3. After heating at 95°C for 5 min, PCR reactions were performed for 32 cycles consisting of heat denaturation (95°C for 45 sec), annealing (60°C for 45 sec), and extension (72°C for 45 sec), a final extension step was carried out at 72°C for 5 min. After NcoI restriction digest, PCR product amplified from normal LT-α allele (10.5 kb) remained undigested. In presence of LT-α variant allele (5.5 kb), 371 bp PCR product were cut into two fragments with 134 and 237 bp lengths (13). A random sample of patients was genotyped twice; no discordances were observed regarding genotyping results.

***Statistical analysis***

Qualitative data were analyzed by chi-square (or Fisher exact) test by the use of SPSS 13 (SPSS Inc.,Chicago, USA) software packages. A *P- *value of <0.05 was statistically significant.

**Table 3:** Characterization of RFLP-PCR primers

Primer sequence	Tm	Product size
TNF-α Forward 5'-AGGCAATAGGTTTTGAGGGCCAT-'3	56.38	107 bp
TNF-α Reverse 5'-TCCTCCCTGCTCCGATTCCG -'3	57.65	
LT-α Forward 5'-CTCCTGCACCTGCTGCCTGGATC -'3	61.4	371bp
LT-α Reverse 5'- GAAGAGACGTTCAGGTGGTGTCAT-'3	56	

## Results

In this study, we aimed to determine TNF-α(-308) and LT-α(+252) polymorphisms in ALL and NHL patient groups with RFLP-PCR. The genotype allele frequencies of TNF-α (-308) and LT-α(+252) polymorphisms in two subjected patients with controls were shown in Table 4 and 5 respectively. According to our data, the frequency of LT-α 5.5 kb allele (hetero and homozygote) in NHL patients and control was 24.6%. vs. 44.8%. There was a statistically significant difference between LT-α+252 polymorphism and these two subjected groups (*P-*value =0.008). The frequency of TNF-2 allele (hetero and homozygote) in NHL patients and control was 14.7% and 18.4%. As a result, there was not any particular association of TNF- α-308 polymorphism in NHL and control group (*P-*value = 0.491). In ALL cases, mostly with Azari origin like NHL patients, there was a statistically significant difference between ALL patients and control group with TNF- α-308 polymorphism (*P-*value =0.005). Here, the allele frequency of TNF-2 in ALL patients and control was 4.3% and 18.4%. In contrast with TNF variant, we could not find any significant association for LT polymorphism between ALL and controls (*P-*value =0.616)

**Table 4:** Allele and genotype frequencies of TNF-α (-308) and LT-α (+252) polymorphisms in non- Hodgkin lymphoma patients and controls

	Allele frequency			Genotype distribution	
Patients Controls	TNF-1	TNF-2	TNF-1**/**1	TNF-1**/**2	TNF-2**/**2
	0/85.3	0/1.5	58(85.3%)	9(13.2%)	1(1.5%)
	0/80.8	0/1.5	105(81.3%)		
Patients Controls	LT-α	LT-α	LT-α	LT-α	LT-α
	(10.5)	(5.5)	(10.5**/**10.5)	(10.5**/**5.5)	(5/5.5**/**5)
	0**/**75.4	0**/**3.3	46(75.4%)	13(21.3%)	2(3.3%)
	0**/**55.2	0**/**9.6	69(55.2%)	44(35.2%)	2(9.6%)

TNF: Tumor necrosis factor. LT-α: Lymphotoxin- alpha

**Table 5:** Distribution and allele frequency of TNF-α (-308) and LT-α (+252) Polymorphisms in ALL patients and controls

	Allele frequency			Genotype distribution	
Patients Controls	TNF-1	TNF-2	TNF-1**/**1	TNF-1**/**2	TNF-2**/**2
	0/85.3	0/1.5	58(85.3%)	9(13.2%)	1(1.5%)
	0/80.8	0/1.5	105(81.3%)		
Patients Controls	LT-α	LT-α	LT-α	LT-α	LT-α
	(10.5)	(5.5)	(10.5**/**10.5)	(10.5**/**5.5)	(5/5.5**/**5)
	0**/**75.4	0**/**3.3	46(75.4%)	13(21.3%)	2(3.3%)
	0**/**55.2	0**/**9.6	69(55.2%)	44(35.2%)	2(9.6%)

TNF: Tumor necrosis factor. LT-α: Lymphotoxin- alpha

## Discussion

NHL and ALL; two main groups of hematological malignancies were affected by some various environmental and individual genetic factors. In this regard, TNF-α(−308) and LT-α(+252) genetic polymorphisms may be relate to susceptibility of these diseases. This research was done to determine the prevalence of TNF-α(-308) and LT-α(+252) polymorphisms in NHL and ALL patients from Tabriz Children Hospital in Northwestern part of Iran. The frequency of heterozygote and homozygote LT-α+252 polymorphism were 21.3% and 3.3% in NHL patients vs. 35.2% and 9.6% in control group respectively. The results of the present study suggest that there was a statistically significant association of LT-α +252 polymorphism with NHL and control group (*P-*value = 0.008). Ibrahim *et al*    (2) have reported that hetero and homozygote frequencies' of LT-α+252 were 29.7%, 10.7% and 42%, 7% in Egyptian NHL patients and healthy controls respectively. They did not find any statistically significant difference of LT-α+252 polymorphism in these groups. Skibola *et al *(23) have reported that LT-α +252 variant genotypes were associated with increased risk of NHL particularly in DLBCL subtype. TNF-α genotyping in NHL patients revealed that 13.2% of patients had TNF-α GA heterozygote and 1.5% had the TNF-α AA homozygote, also in control group these were 16.5% and 1.5%, respectively. No significant difference were found for the TNF-α variant in NHL and control group (*P-*value =0.491). This was in accordance with Rothman *et al *(17), and Cerhan *et al* (16) who found an association only with DLBCL but not in other subtypes; However, results from Purdue *et al *(10) and Jrad *et al *(18) were not similar to this study. Therefore, our results suggested that in NHL and healthy control group mostly with Azari ethnicity there is a significant relationship only in the LT-α variant. In a study in Wales and Germany  (20), the prevalence of TNF and LT alleles were different and there was a significant association of TNF-a in NHL and controls in Wales (10). Skibola *et al (*23) have reported that the frequencies of TNF-α GA and TNF-α AA genotypes in NHL and control groups from eight European countries, Canadian and US were 26%, 3% and 23%, 3% respectively. In ALL patients; TNF-α (GA allele) allele frequency was less than control group and we did not have any TNF-α AA variant in patients. This alleles frequency was different in German population (13). Although the frequencies of TNF-α-GA and AA genotypes in ALL patients was less than from Takeuchi *et al *(12) but here, we could find a statistically significant relationship of TNF-α -308 polymorphism was found between ALL patients and control group (*P-*value= 0.005); Oppositely, Takeuchi *et al *(12) and Kidas *et al *(13) did not find any statistically significant relationship between TNF-α -308 polymorphism and ALL patients. 

At the end, the allele frequencies of LT- α AG and LT- α GG genotypes in ALL patients were 38.6% and 5.7% and also 33.8% and 9.2% in control group. No statistically significant association was found for LT-α +252 polymorphism among ALL and controls (*P-*value = 0.616). In accordance with our result, Zhao *et al* (21) did not find a statistically significant relationship with LT-α+252 polymorphism and ALL patients. However, Stanulla *et al *(22) Takeuchi *et al *(12) did not report this result from their researches. These data suggest that there is a remarkably meaningful relationship only in TNF-α-308 genetic polymorphism with patients who suffering from childhood ALL and control group. Previous studies from Zhao *et al* (21) in Chinese and Takeuchi S(12) in German people have shown that there was not any relationship of TNF-α-308 and LT-α+252 with ALL. Several studies have reported different prevalence of TNF-α-308 and LT-α+252 polymorphisms and their association with susceptibility to NHL and ALL, however, results are different. Some authors described a poor prognosis with high producer variant; others could not find such association. Seidemann *et al *  (20) indicated that there was no association between genotypes and clinical characteristics of NHL in patients from Austria, Germany and Switzerland and TNF-genetic polymorphisms are not a main prognostic factor for pediatric and adolescent NHL cases  (20). A recent study from Skibola *et al *  (23) which analyzed the risk of NHL in various ethnic groups indicated that TNF-α (–308A) allele are related with an increased risk in non-Hispanic white and blacks but it has reduced risk of all NHL in Asian populations**.**

According to the Cerhan *et al *  (24) results, individuals who carried TNF-GA genotype was not associated with risk or susceptibility to NHL whereas TNF- AA genotype have a meaningful association with NHL. Both polymorphisms are associated with increased production of TNF and LT cytokines (2, 22). TNF has a powerful anti-tumoral activity but if cytokines remain in body for a long time, they lose their anti tumor activity. Until now, how overproduction of TNF and LT can influence the clinical course of malignancy is unclear (7) but increased TNF and LT levels for long time in body may impair efficiency of antitumor cellular immune responses (14). 

However, the detailed pattern of NHL heritability remains unclear (19) because large population study is needed to evaluate the relationship or risk of NHL. Also, functional studies are needed to explore the effects of these polymorphisms in subjected malignancies.

## Conclusion

The present results reported the prevalence of TNF-α (-308) and LT-α (+252) genetic polymorphisms in NHL, ALL patients and normal control group in Northwestern part of Iran but these are different from other studies which are performed in various populations. These different allele frequencies may be related to difference in ethnic and populations and selected samples.

According to our data, there are statistically meaningful differences of TNF-α-308 polymorphism in ALL and LT-α+ 252 variant in NHL patients. Several studies examined the relationship of TNF α-308 and LT α+252 polymorphisms with susceptibility to NHL and ALL diseases. Because both variant alleles of TNF-α and LT-α have shown to correlate with elevated plasma levels; evaluation of TNF-α-308 and LT-α+ 252 genetic polymorphisms will be useful in determination of susceptibility to NHL and ALL diseases. However, additional works with high population are needed to clarify the genetic and biologic basis of these malignancies.
